# Supplementary material for: COZOID: contact zone identifier for visual analysis of protein-protein interactions
Source: BMC Bioinformatics. 2018 Apr 6;19:125. doi: 10.1186/s12859-018-2113-6 (PMC5889581; doi:10.1186/s12859-018-2113-6)
Supplement: Supplementary file 2 — Software build. Executable binary file of the software tool. (ZIP 118,784 kb) [file 12859_2018_2113_MOESM2_ESM.zip › cozoid/java/docs/beansbinding-1.2.1-doc.zip!/org/jdesktop/beansbinding/package-summary.html]

org.jdesktop.beansbinding (Beans Binding)


---


|  |  |  |
| --- | --- | --- |
| **Overview** \| **Package** \| Class \| **Use** \| **Tree** \| **Deprecated** \| **Index** \| **Help** | |  |
| PREV PACKAGE   **NEXT PACKAGE** | **FRAMES**    **NO FRAMES**     **All Classes** |  |


---

## Package org.jdesktop.beansbinding

Provides support for defining properties and creating bindings between
sets of two properties.

**See:**
  
          **Description**

| **Interface Summary** | |
| --- | --- |
| **BindingListener** | `BindingListeners` are registered on `Bindings` or `BindingGroups` to listen for changes to the state of `Bindings` |
| **PropertyStateListener** | `PropertyStateListeners` are registerd on `Property` instances, to be notified when the state of the property changes. |

| **Class Summary** | |
| --- | --- |
| **AbstractBindingListener** | An abstract subclass of `BindingListener` that simplifies writing `BindingListeners` by allowing you to extend this class and re-implement only the methods you care about. |
| **AutoBinding<SS,SV,TS,TV>** | An implementation of `Binding` that automatically syncs the source and target by refreshing and saving according to one of three update strategies. |
| **BeanProperty<S,V>** | An implementation of `Property` that uses a simple dot-separated path syntax to address Java Beans properties of source objects. |
| **Binding<SS,SV,TS,TV>** | `Binding` is an abstract class that represents the concept of a binding between two properties, typically of two objects, and contains methods for explicitly syncing the values of the two properties. |
| **Binding.SyncFailure** | `SyncFailure` represents a failure to sync (`save` or `refresh`) a `Binding`. |
| **Binding.ValueResult<V>** | Encapsulates the result from calling `Binding.getSourceValueForTarget()` or `Binding.getTargetValueForSource()`, which can either be a successful value or a failure. |
| **BindingGroup** | `BindingGroup` allows you to create a group of `Bindings` and operate on and/or track state changes to the `Bindings` as a group. |
| **Bindings** | A factory class for creating instances of the concrete `Binding` implementations provided by this package. |
| **Converter<S,T>** | `Converter` is responsible for converting a value from one type to another. |
| **ELProperty<S,V>** | An implementation of `Property` that allows Java Beans properties of source objects to be addressed using a simple dot-separated path syntax within an EL expression. |
| **ObjectProperty<S>** | An immutable, read-only, `Property` implementation whose `getValue` method returns the source object that it is given. |
| **Property<S,V>** | `Property` defines a uniform way to access the value of a property. |
| **PropertyHelper<S,V>** | An abstract subclass of `Property` that helps with the management of `PropertyStateListeners` by implementing the methods for adding, removing, and getting listeners. |
| **PropertyStateEvent** | An event characterizing a change in a `Property's` state for a particular source object. |
| **Validator<T>** | `Validator` is responsible for validating the value from the target of a `Binding`. |

| **Enum Summary** | |
| --- | --- |
| **AutoBinding.UpdateStrategy** | An enumeration representing the possible update strategies of an `AutoBinding`. |
| **Binding.SyncFailureType** | An enumeration representing the reasons a sync (`save` or `refresh`) can fail on a `Binding`. |

| **Exception Summary** | |
| --- | --- |
| **PropertyResolutionException** | `PropertyResolutionExceptions` can be thrown at various points in the life cycle of a `Property`. |

## Package org.jdesktop.beansbinding Description

Provides support for defining properties and creating bindings between
sets of two properties.

### Properties

A property is defined by creating an instance of a concrete implementation
of the `Property` class. This package
provides two concrete `Property` implementations of interest:
`BeanProperty` allows Java Bean properties
to be addressed by providing their path as a String.
`ELProperty` allows Java Bean properties
to be addressed similarly and then be used in various ways in an EL expression.
A simple `BeanProperty` that refers to a "firstName" Java Bean property,
might look like:

```
    BeanProperty firstNameP = BeanProperty.create("firstName");
```

An `ELProperty` to combine the "firstName" and "lastName"
properties of a Java Bean into a full name, might look like:

```
    ELProperty fullNameP = ELProperty.create("${firstName} ${lastName}");
```

These property objects can then be used to operate on a Java Bean having
the properties of interest. For example:

```
    // prints the first name on the person object
    System.out.println(firstNameP.getValue(person));

    // prints the full name of the person object
    System.out.println(fullNameP.getValue(person));

    // sets the first name of the person object to "Duke"
    firstNameP.setValue(person, "Duke");

    // listen for changes to the person's full name
    fullNameP.addPropertyStateListener(person, listener);
```

Both of these property implementations are designed to work with bean
properties that follow the Java Beans specification. Sometimes, however,
a property of interest on a bean outside of your control may not be exposed
in the correct way, and you want to adapt it for use in binding.
Other times you may want to extend the set of properties that a bean
exposes, simply for use in binding. These cases are provided for by
the org.jdesktop.beansbinding.ext
package.

### Bindings

A binding is created between two `Property` instances, and the objects
on which the `Property` objects should operate, by creating an instance
of a concrete implementation of the `Binding` class. Once the binding is
realized, by a call to the `bind` method, a `Binding` starts
tracking changes to the properties on both ends, and a typical `Binding`
implementation will sync the properties with each other based on on some defined
strategy.

This package provides one concrete subclass of `Binding` called
`AutoBinding` which syncs the properties based
on a configurable update strategy. `AutoBindings` are created by
calling one of the static `createAutoBinding` methods in the
`Bindings` class. For example:

```
    BeanProperty firstNameP = BeanProperty.create("firstName");
    BeanProperty textP = BeanProperty.create("text");
    Binding binding = Bindings.createAutoBinding(READ_WRITE, person, firstNameP, jTextField, textP);
    binding.bind();
```

Before a value from a source property is set on a target property, it passes
through an optional `Converter` to convert
it between the source type and target type. Before a value passed from a
target property back to a source property, it passes first through the
optional `Converter` and then through an optional
`Validator`, which can reject invalid values.

---


|  |  |  |
| --- | --- | --- |
| **Overview** \| **Package** \| Class \| **Use** \| **Tree** \| **Deprecated** \| **Index** \| **Help** | |  |
| PREV PACKAGE   **NEXT PACKAGE** | **FRAMES**    **NO FRAMES**     **All Classes** |  |


---
